# Differential Features of Cholecystokinin-Releasing Peptides Derived from Food Proteins: Peptide Length, Amino Acid Composition and Primary Structure; Analysis of Currently Identified Peptide Sequences

**DOI:** 10.3390/ijms262211065

**Published:** 2025-11-15

**Authors:** Giovanni Tulipano

**Affiliations:** Unit of Pharmacology, Department of Molecular and Translational Medicine, University of Brescia, 25123 Brescia, Italy; giovanni.tulipano@unibs.it; Tel.: +39-03-0371-7510

**Keywords:** bioactive peptides, food proteins, identification and characterization, cholecystokinin, enteroendocrine hormones, enteroendocrine cells, CCK-releasing peptides

## Abstract

The mouse enteroendocrine cell line STC-1 has been widely used to investigate the effects of dietary protein-derived peptides on cholecystokinin (CCK) secretion. The studies have also addressed the question of whether specific structural features of a given peptide chain may be related to higher secretagogue activity with respect to others, but a detailed structure–activity relationship in CCK-releasing peptides has not yet been reported. The aim of this study was to list the currently available CCK-releasing peptide sequences; to draw conclusions about the role played by peptide length, peptide amino acid composition and peptide amino acid sequence in differentiating their secretagogue activity; and to highlight the physicochemical properties and sequence motifs shared by the active peptides, and any possible differential feature between CCK-releasing peptides and ineffective peptides. To this end, a method was applied consisting of the fractionation of peptide sets into subsets and the comparison between paired subsets of active and inactive peptides. A few distinctive structural features related to CCK-releasing activity were highlighted for each subset. Actually, minor changes in the primary structure can make the difference between active and inactive peptides, as suggested by previous studies. Hence, the chance of predicting the activity of a peptide that has never been tested in vitro using reference structures must still be considered to be low.

## 1. Introduction

It is recognized that the intake of dietary proteins exerts a greater suppressive effect on hunger and energy intake compared with an isoenergetic intake of fat and carbohydrates. This effect on satiety is partially dependent on the release of anorexigenic gut hormones in response to the detection of protein digestion products by the gut wall. To this regard, nutrient sensing by enteroendocrine cells facing the gut lumen is known to be mediated by specific cell surface receptors, mainly G-protein-coupled receptors and membrane transporters, so-called nutrient-sensing receptors.

The main peptidic hormones released into circulation by the enteroendocrine cells in the gut in response to an oral nutrient load include cholecystokinin (CCK), Tyrosine-Tyrosine (PYY), oxyntomodulin, glucagon-like-peptide-1 (GLP-1) and glucose dependent insulinotropic peptide (GIP). Distinct subtypes of enteroendocrine cells differ in the hormones they can release and in their localization along the GI tract. Among gut hormones, CCK is secreted by I cells in the proximal small intestine in response to luminal peptides and lipids and cooperates with the signaling networks in regulating appetite, food intake, gastric emptying, pancreatic enzyme secretion and gall bladder contraction [1,2,3,4].

In the last two decades, the mouse enteroendocrine cell line STC-1 has been widely used to investigate the effects of dietary proteins and peptides on CCK- and glucagon-like peptide-1 (GLP-1) secretion. These studies have also addressed the question of whether the specific structural features of a given peptide chain may be related to higher secretagogue activity with respect to others. A number of authors have reported on the isolation and identification of peptide fragments linked to a strong induction of hormone release, either CCK, or GLP-1 or both, between the products of enzymatic simulated gastrointestinal digestion (SGID) of distinct dietary proteins [5,6,7,8,9,10,11,12,13,14,15,16,17]. Despite some studies highlighting a few structural characteristics shared by peptides endowed with high secretagogue activity, a detailed structure–activity relationship among CCK- or GLP-1-releasing peptides has not been reported yet. In fact, according to the current data available, this outcome may hardly be reachable.

In this regard, some factors may significantly contribute to expanding the heterogeneity of receptor ligands: briefly, a number of different nutrient-sensing receptors are involved in mediating the response of enteroendocrine cells to proteins and their digestion products; these receptors are able to detect both free amino acids and peptides; and finally, when limited to those observed using STC-1 cell cultures, the active concentrations of peptide ligands are relatively high (approx. 0.1–1 mM), suggesting low affinity ligand–receptor interactions [1,4,18].

Nevertheless, research works reporting on food-protein-derived peptides acting as inducers of CCK release in STC-1 cell cultures in similar experimental conditions have accumulated in the last decade. These works make it possible to file a sizable number of peptide sequences acting as CCK-releasing factors, and to compare them to a set of peptide sequences which were revealed to be ineffective in the same trials [5,6,7,8,9,10,11,12,13,14,15,16].

The aim of this study was to list the currently available CCK-releasing peptide sequences identified using STC-1 cell cultures; to draw conclusions about the role played by peptide length, peptide amino acid composition and peptide amino acid sequence in differentiating their secretagogue activity; and to highlight the physicochemical properties and sequence motifs shared by the active peptides, if any, as well as any possible differential features between CCK-releasing peptides and ineffective peptides.

## 2. Results

**Figure 1:**
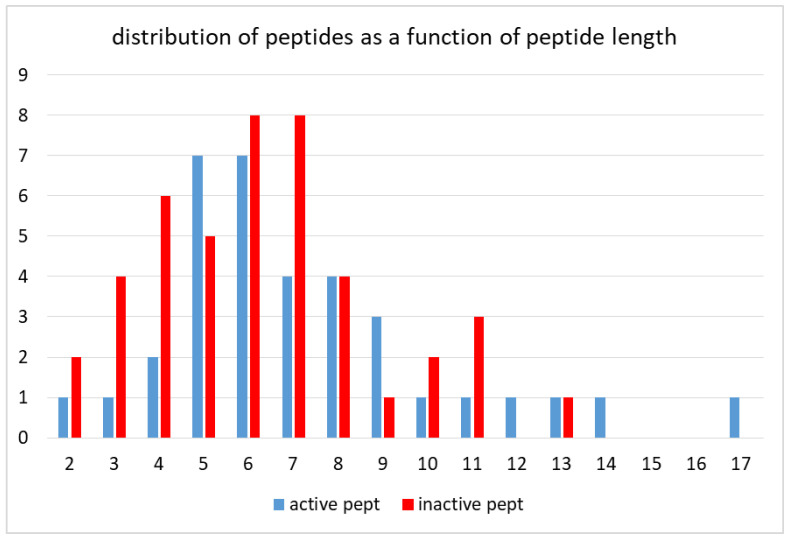
Frequency distribution of peptides as a function of peptide size. The number of peptides of the same length (from 2 to 17 amino acids) is shown.

The “active peptide” and the “inactive peptide” set included 35- and 44-peptide sequences, respectively. The peptide length ranged from 2 amino acids up to 17 amino acids. The analysis of the frequency distribution as a function of peptide size (Figure 1) revealed that most of the peptides were five to eight amino acids long: 62.85% of active peptides vs. 56.8% of inactive peptides. As to the two tails of the distribution, the relative amount of longer peptides (9 to 17 amino acids) was 25.7% of active peptides versus 15.9% of inactive peptides, whereas the relative amount of shorter peptides (2 to 4 amino acids) was 14.8% of active peptides versus 27.2% of inactive peptides.

### 2.1. First Subset: Peptide Length from Two to Four Amino Acids

The pI values ranged from 3.7 to 9.1 in active peptides, and 3.8 up to 8.6 in inactive peptides. The comparison of the amino acid composition between the two groups revealed that L-Phe was exclusively present in active peptides (two out of four peptides), whereas the acidic amino acid L-Glu was found only in inactive peptides (four out of twelve peptides). The sole acidic peptide promoting CCK release (pI 3.7) contained L-Asp in place of L-Glu (Table 1).

Apart from the presence of specific amino acids, the secretagogue activity may be related to physicochemical properties conferred by the side chains and shared by some amino acids. To this end, the color scheme used in this work (see Section should help with visualizing the similarities and differences between the two groups of peptides in terms of amino acid composition by highlighting the presence of aliphatic/aromatic, polar (with amide or hydroxyl groups), charged (basic or acidic), Sulfur-containing, or peculiar side chains.

The analysis showed that the active and the inactive peptides differ significantly in their N-terminal ends, with large hydrophobic (aliphatic) side chains in the second group versus L-Arg and L-Pro in the first group.

As to the comparison of the amino acid sequences, the alignment between the two sequences RALG (active) and RYLG (inactive) showed that the presence of L-Arg is not enough to confer CCK-releasing activity. Specifically, Santos-Hernandez and coworkers showed that one amino acid change within this short sequence (tiny-aliphatic replaced by large-aromatic amino acid with higher level of hydrophilicity), can cause a loss of activity [8]. Finally, both groups of peptides contained L-Pro, which was located at the N-terminus in active peptides versus inner positions or the C-terminus in inactive peptides (Table 1).

Table 2 shows the alignment between a subgroup of active peptide sequences and the sequence RFYGPV. This hexapeptide matches the C-terminal end of human osteocalcin and is an allosteric ligand to GPRC6A receptor, a nutrient-sensing receptor in enteroendocrine cells [19]. The comparison revealed a similarity in amino acid composition (the shared amino acid being L-Arg, L-Phe, L-gly and L-Pro), the presence of L-Arg at the N-terminus and/or L-Phe in second position, and an aliphatic amino acid at the C-terminal end.

### 2.2. Second Subset: Peptide Length from Nine to Seventeen Amino Acids

The pI values ranged from 3.29 to 12.98 in active peptides, and 5.07 to 10.82 in inactive peptides. The H_R_ values ranged from 5.32 to 37.3, and 7.5 to 26.23, respectively, (Table 3).

In order to compare the amino acid composition between the two groups of peptides, we considered the total amount of each amino acid in the active sequences versus the inactive sequences, expressed as a percentage of the sum of all amino acid moles in the respective group.

The comparison suggested a higher content of L-Leu, L-Val, and L-Asp in the active sequence pool versus a higher content of L-Gln and L-Pro in the inactive sequence pool. Specifically, the presence of multiple units of L-Pro scattered along the sequence and flanked by L-Gln and/or L-Gly, was the distinctive trait of four out of seven inactive peptides. Remarkably, L-Pro and L-Gly are both regarded as conformationally special amino acids. The content of aromatic amino acids was low in both groups of peptides. Finally, all the active peptides but one contained L-Asp and/or L-Glu whereas five out of seven inactive peptides did not contain any acidic amino acid (Table 4).

The analysis suggested the separation of the active peptides into two subgroups: the first one includes five peptides with low pI value joined with a high H_R_ value. The second subgroup includes four peptides containing two, three or five basic amino acids, respectively, scattered along the sequence.

Among the acidic peptides, three peptides contained multiple L-Glu and/or L-Asp residues (five or six residues in each peptide). The alignments of these peptides showed extensive sequence similarities. Apart from the clusters of acidic amino acids, the common traits included one L-Thr, an aromatic amino acid (L-Phe or L-Tyr) and, finally, amino acids with aliphatic side chains (mainly L-Leu and L-Val) flanking the acidic clusters. The alignment of the remaining two peptides, which contained one and two acidic residues, respectively, showed a scrambled sequence of six amino acids, including L-Glu, setting aside the insertion of a second acidic residue in one peptide (Table 5).

Among basic peptides, the longest sequence matched the soy-beta conglycinin 51–63 peptide [14]. The alignments showed some similarities between this sequence and two other distinct sequences, so that they all may be gathered into a subgroup named as soy-beta conglycinin 51–63 peptide-related subgroup. The shared traits were: aliphatic amino acid at the N-terminus (L-Val or L-Leu); basic amino acids (L-Arg or L-Lys) scattered along the sequence; prevalence of amino acids with hydrophilic side chains (polar- or charged-) in the C-terminal half of the peptide; and presence of amino acids with a hydroxyl group in their side chains at the C-terminus or in the second position starting from the C-terminus. Finally, a second alignment analysis highlighted the presence of an inverted sequence of five amino acids shared by the peptides endowed with pI values of 8.63 and 8.03, respectively, (Table 6).

### 2.3. Third Subset: Peptide Length from Five to Eight Amino Acids

**Figure 2:**
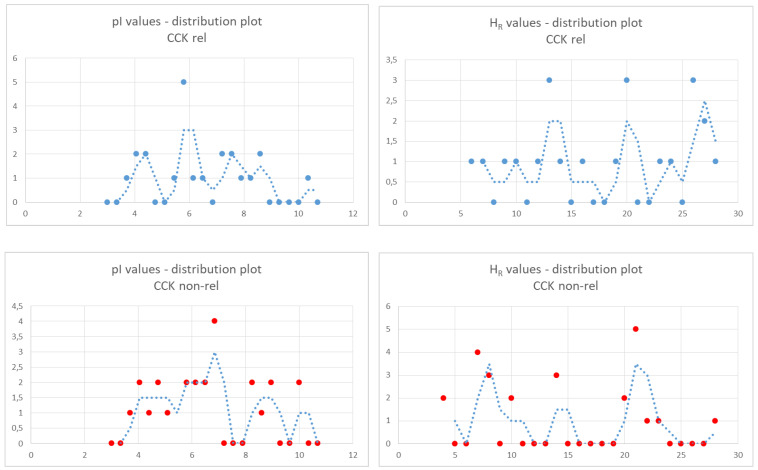
Distribution of peptides according to their pI or H_R_ values. For each set of peptides (active- vs. inactive peptides), the values were grouped into bins and the number of elements into each bin was shown by using dots in the distribution plots. Trend lines are shown (dotted lines). The bin size was 0.35 and 1.0 units for the pI plot and H_R_ plot, respectively.

The number of peptides in the two groups (22 active vs. 25 inactive peptides) (Table 7 and Table 8, respectively) made it possible to analyze the distribution of pI and H_R_ values. To this end, we grouped the values into bins and displayed the number of elements in each bin by using dots in the distribution plots. The bin sizes were 0.35 and 1.0 units for the pI plot and H_R_ plot, respectively, and were calculated as follows: (max value − min value)/(number of values). Trend lines are also shown. The distribution plots of the pI values were similar between the two groups. On the other hand, the distribution of H_R_ values showed a greater number of values lower than 10 in the inactive peptide group versus a greater number of values higher than 25 in the active peptide group, suggesting that a marked hydrophobicity of a five- to eight-amino-acid-long peptide might be a positive marker of CCK-releasing activity (Figure 2).

Finally, assuming normal distribution of values, we calculated the mean values of pI and H_R_ in the two groups. We observed similar mean pI values, whereas the mean H_R_ value tended to be lower in inactive peptides compared to active peptides (pI: 6.2 ± 1.7 vs. 6 ± 2; H_R_: 17.9 ± 7.6 vs. 14.1 ± 7.4, as the mean ± SD of 22 vs. 25 values; unpaired *t* test, *p* < 0.1), in agreement with the conclusion of the distribution analysis.

We used a bioinformatic tool for peptide solubility prediction, wherein solubility was defined in PROSO II as a sequence that was transfectable, expressible, secretable, separable and soluble in an *E. coli* system (see Section 4 for details). Most peptides were predicted as weakly soluble (59% of active vs. 68% of inactive peptides). In total, 23% of active peptides and 20% of inactive peptides were predicted as unlikely to be soluble. The remaining peptides were likely soluble (18% of active vs. 8% of inactive peptides).

The comparison of the cumulative amino acid composition between active and inactive peptides suggested a higher relative content of L-Leu, L-Asp, L-Arg and L-Tyr and a lower relative content of L-Gln, L-Gly and L-Thr in the active peptide sequence group versus the inactive sequence group (Table 9). Moreover, we might want to remark that the active peptides in this subset contained a greater percentage of aromatic amino acids and L-Pro and a lower percentage of L-Lys, L-Glu and L-Thr compared to the active peptides in the second subset (meaning active peptides longer than nine amino acids) (Table 9 versus Table 4).

The relative content of L-His, L-Met, L-Phe, L Val, L-Ile, L-Ser, L-Lys and L-Pro was similar in active versus inactive peptides. Restricted to these amino acids, we examined their flanking amino acids within all the active peptide sequences, and then we listed the C-terminal and N-terminal dipeptides and the tripeptides containing each amino acid (Table 10). We did not include the di- or tripeptides that were also found in inactive peptide sequences. The outcome was a list of short amino acid motifs which may be related to CCK-releasing activity. L-His was found at the C-terminal end or flanked by amino acids with hydrophobic side chains in tripeptides. L-Phe was mainly found at internal positions, whereas L-Ser was at terminal positions (N-terminal or C-terminal end, indifferently). L-Ile was linked to an aromatic amino acid and/or L-Arg in tripeptides, and to L-Pro in N-terminal or C-terminal dipeptides. Finally, the relative content of L-Pro and L-Val was high in both active and inactive peptide sequences and the related amino acid motifs were heterogeneous in both groups.

In order to analyze the amino acid sequences to find possible elements shared by active peptides and possible differential elements between active and inactive peptides, the sequences were separated into three subgroups according to their pI values: low pI values (acidic), high pI values (basic), and intermediate pI values.

The relative amount of acidic peptide sequences was 22.73% of active peptides versus 20% of inactive peptides, respectively. The H_R_ values of the inactive peptides did not exceed 10, whereas four out of five active peptides showed H_R_ values higher than 13 and two values that were near to 20, suggesting that a low pI value along with hydrophobicity may be a positive marker of CCK-releasing activity (Table 7 and Table 8).

The alignment of the five active sequences highlighted some common traits: specifically, the presence of a core made of aliphatic amino acids (mainly, L-Leu) and L-Asp (in one peptide replaced by L-Glu) was joined with charged amino acids at the C-terminal end. Moreover, the alignment of the active peptide DLVDK with the inactive peptide LGVDE suggested that the insertion of L-Gly might be related to a reduction in CCK-releasing activity. It is worth remarking that the pI and H_R_ values of these peptides were quite similar (Table 11).

The relative amount of basic peptide sequences was 18.2% of active peptides versus 28% of inactive peptides, respectively. In both groups, the pI values ranged approximately from 8 to 10. The H_R_ values varied significantly in both subgroups, although hydrophobicity was the prevailing character in active peptides (Table 7 and Table 8).

The alignment of four active sequences revealed a common structural motif centered on L-Arg and an aromatic amino acid (L-Tyr or L-Trp) at the N-terminal side of the peptide sequence (Table 12, panel A). Then, in order to identify possible differential amino acid motifs between active and inactive peptides, we examined pairs of peptide sequences endowed with similar pI and HR values. The alignments shown in Table 12 (panel B) confirmed that the simultaneous presence of an aromatic amino acid and L-Arg at the N-terminal side was necessary for CCK-releasing activity. Indeed, the peptide YPWQRF was inactive, despite being the richest one in aromatic amino acids at the N-terminal end, and the removal of the N-terminal L-Trp from WIRGCRL caused a loss of activity despite the N-terminal L-Arg in the truncated peptide (see also ref. [8]). However, the presence of L-Lys as a basic amino acid and L-Phe as an aromatic amino acid at the N-terminal side was not related to any secretagogue activity in basic peptides (Table 12, panel B). It is worth remarking that the latter note has not been extended to all the subsets of five- to eight-amino-acid-long peptides.

The relative amount of peptides endowed with intermediate pI values was 59% of active peptides versus 48% of inactive peptides. The pI values ranged from 5.4 to 7.6 in the active peptide subset and 5.0 to 6.8 in the inactive peptide subset. The number of peptides in both subsets made it possible to analyze the peptides with pI higher than 6.0 (neutral) and the peptides with pI lower than 5.9 (mildly acidic) separately.

Among the neutral peptides (Table 13), the pI values of active sequences ranged from 6.5 to 7.6 and exceeded 7.2 in four out of six sequences, whereas the pI value of inactive sequences never exceeded 6.8. The related H_R_ values ranged from 11 to 27 units in active sequences and 7 to 23 in inactive sequences, so that the most hydrophobic sequences were active, whereas the most hydrophilic sequence was inactive. The cumulative amino acid composition was similar in neutral active peptides versus neutral inactive peptides. The main differences were the higher amount of polar amino acids with amide groups in inactive sequences, and the presence of L-Met and L-Tyr only in active sequences. These remarks suggest that CCK-releasing activity was most likely related to the presence of differential amino acid sequence motifs rather than specific amino acid residues. Specifically, the two groups differed significantly in the molecular size/degree of hydrophilicity of the N-terminal residues and the third residue starting from the N-terminal end. Finally, the alignment of the active peptide sequences revealed two coupled structural elements found across four sequences: L-His at position 5 or 6 joined with an aliphatic branched-chain amino acid at position 3 or 2 from the N-terminal end (L-Leu in three peptides and L-Val in the octapeptide). In three out of four sequences, L-His was the C-terminal amino acid residue (Table 13).

As to the mildly acidic peptides, the pI values ranged from 5.4 to 5.8 for active sequences and 5.1 to 6.0 for inactive sequences. The H_R_ values of three active peptides exceeded 27 units, whereas 24 units was the highest value among the inactive peptides. Both groups contained two peptides with higher degrees of hydrophilicity and similar H_R_ values (lower than 10 units). The main differences in the cumulative amino acid composition were the higher contents of polar amino acids with an amide group in the inactive sequences, the same as was observed in neutral peptides, and the presence of L-Arg, L-His, L-Tyr and L-Trp only in the active sequences (Table 14). Finally, the alignment of the active peptide sequences did not show any common trait shared by the majority of peptides; nevertheless, it highlighted some amino acid motifs shared by coupled peptides and not found in the inactive peptide sequences: L-Pro-L-Leu-aromatic amino acid (L-Trp or L-Phe) versus L-Pro-L-Leu-L-Asn in the inactive peptide; L-Val-L-Thr-(L-Gly)-L-Val versus L-Val-L-Thr-L-Gln in the inactive peptide; L-Arg (N-terminal)-...-...-...-...-...-L-Asp (or L-Glu), not found in inactive peptides (Table 14).

### 2.4. Description of Physicochemical Properties of Peptides Using Two Sets of Amino Acid Scalar Descriptors

#### Peptide Length from Five to Eight Amino Acids

The use of line graphs helped to visualize differences between active and inactive peptides in scalar descriptors related to hydrophobic character, charge concentration and volume of the amino acid residues from the N-terminal to the C-terminal end.

**Figure 3:**
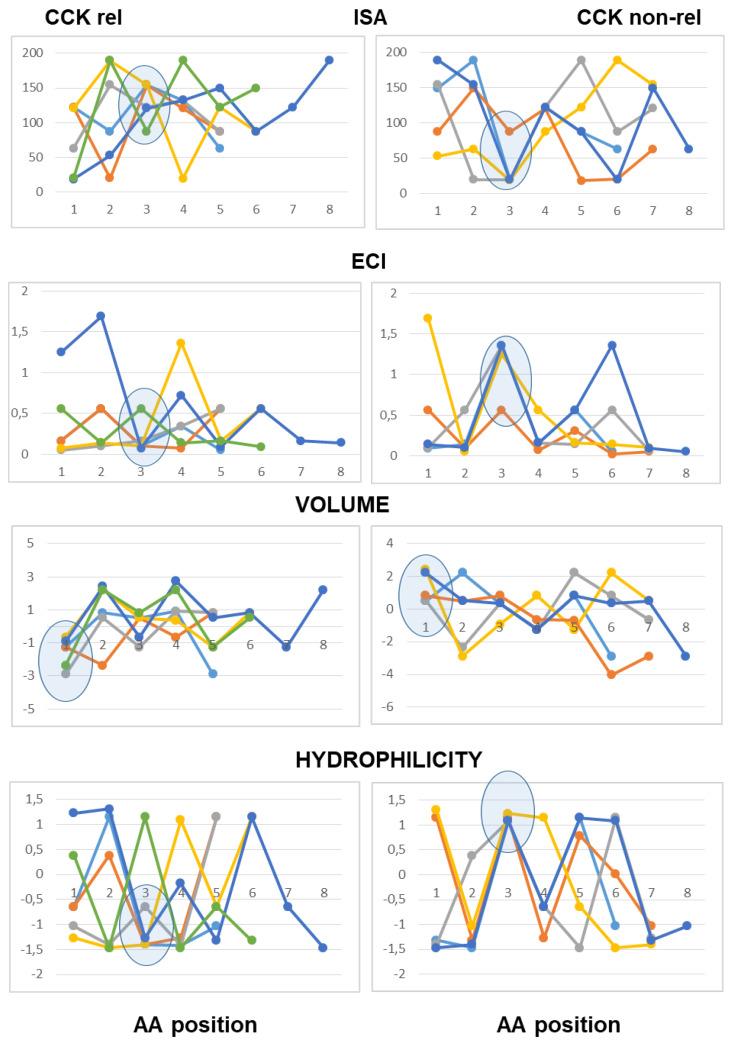
Five- to eight-amino-acid-long peptides (pI range: 6.1–7.9). Each peptide is described by a series of data points connected by straight lines. Data points corresponded to the scalar descriptor values of the amino acids from the N-terminal (position 1) up to the C-terminal position within the peptide. Two scalar descriptors have been used: the scale developed by Collantes and Dunn (isotropic surface area, ISA and electronic charge index, ECI) and the “Volume” and “Hydrophilicity” scale (see Section 4 for details).

As to peptides with neutral pI values (6.1–7.9), the analysis highlighted a difference at the third position from the N-terminal end according to both scales of descriptors, with hydrophobic side chains and reduced charge concentration in active peptides versus polar or charged side chains in inactive peptides. According to the Barley scale, active peptides also differed from inactive peptides in the molecular size of N-terminal amino acids (Figure 3).

**Figure 4:**
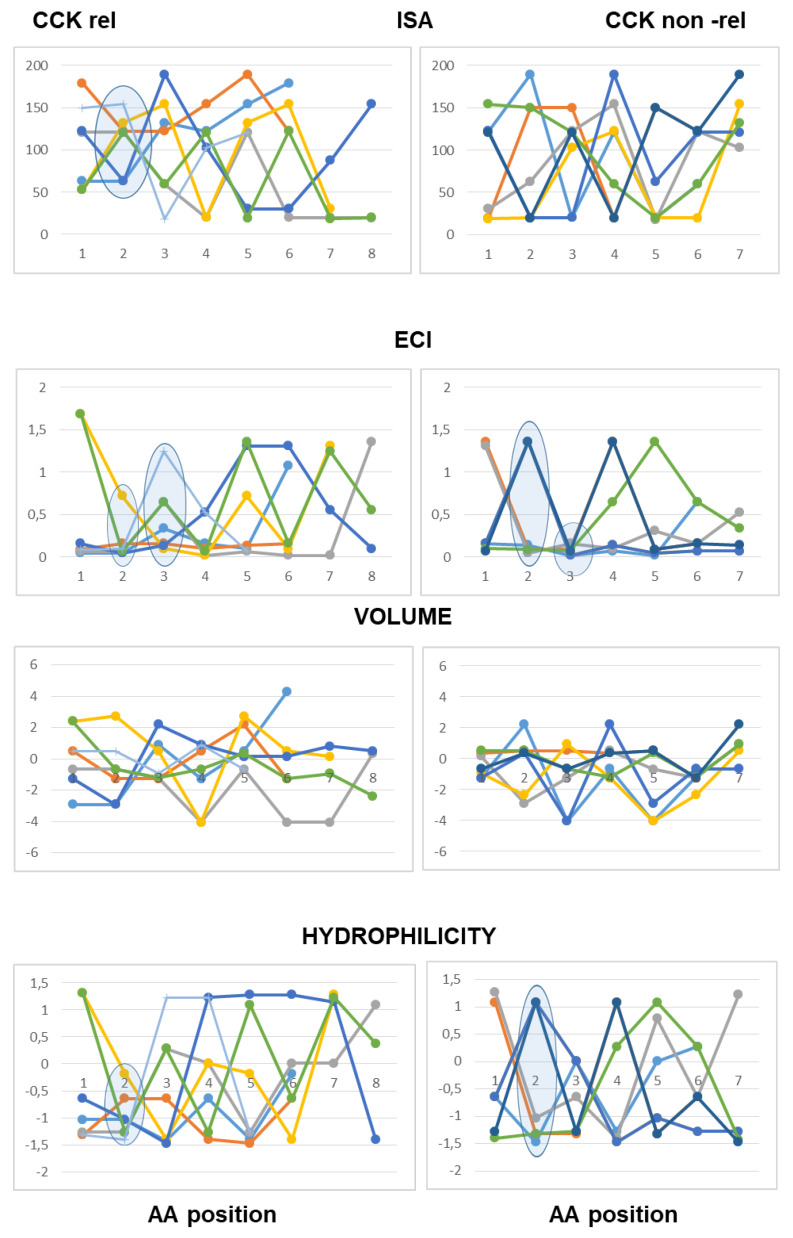
Five- to eight-amino-acid-long peptides (pI range: 5.1–6.0). Each peptide is described by a series of data points connected by straight lines. Data points correspond to the scalar descriptor values of the amino acids from the N-terminal (position 1) up to the C-terminal position within the peptide. Two scalar descriptors have been used: the scale developed by Collantes and Dunn (isotropic surface area, ISA and electronic charge index, ECI) and “Volume” and “Hydrophilicity” scale(see Section 4 for details).

As to the peptides endowed with pI values lower than 6.0 (mildly acidic), the use of the Collantes and Dunn scalar descriptors highlighted some structural features found only in inactive peptide sequences: specifically, the occurrence of amino acids with very high or very low ISA values (out of the range of 60 to 130) at the second position from the N-terminus, and the occurrence of amino acids with high ECI values (higher than 0.72) at the second position joined with amino acids with very low ECI values (lower than 0.1) at the third position from the N-terminus. The use of the Barley scalar descriptors confirmed the relevance of the hydrophobic character of the side chain at position 2, in agreement with the previous scale, whereas it did not suggest a distinct trait related to the molecular size of active peptides versus inactive peptides (Figure 4).

**Figure 5:**
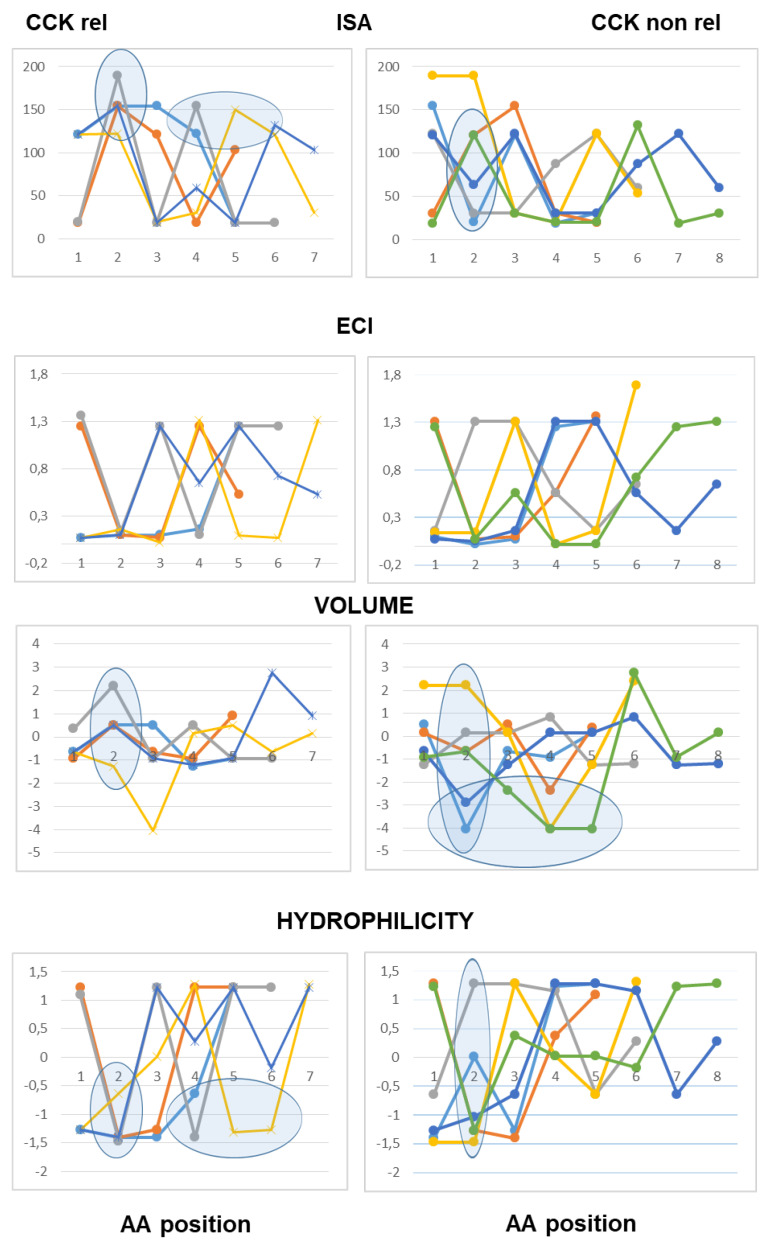
Five- to eight-amino-acid-long peptides (pI < 5.0). Each peptide is described by a series of data points connected by straight lines. Data points correspond to the scalar descriptor values of the amino acids from the N-terminal (position 1) up to the C-terminal position within the peptide. Two scalar descriptors have been used: the scale developed by Collantes and Dunn (isotropic surface area, ISA and electronic charge index, ECI) and “Volume” and “Hydrophilicity” scale (see Section 4 for details).

As to the acidic peptides (pI lower than 5.0), the comparison of the line graphs obtained using the Barley scalar descriptors suggested remarking on some peculiar traits that may contribute to differentiating active and inactive peptides. The volume values varied over a wider range in the inactive peptide sequences compared to the active peptide sequences. Specifically, the insertion of tiny amino acids (L-Gly, L-Ala and L-Ser) along the sequence was related to lack of CCK-releasing activity. Moreover, according to both scales of descriptors, the presence of amino acids with large hydrophobic side chains (aromatic or aliphatic) at position 2 from the N-terminal end was required to confer CCK-releasing activity, in that small side chains and/or hydrophilic side chains at the second position were seen only in inactive peptides. Of course, this trait was not enough to confer activity (Figure 5).

**Figure 6:**
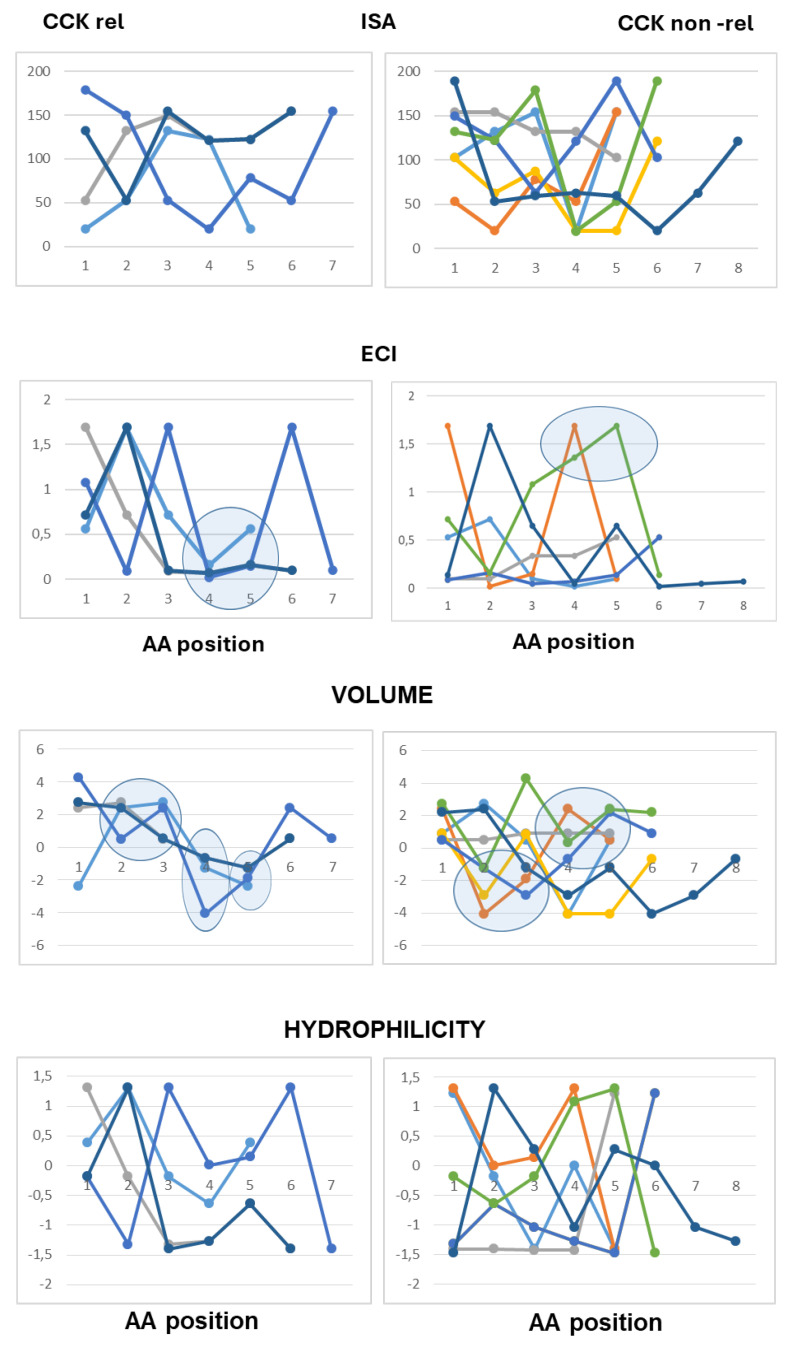
Five- to eight-amino-acid-long peptides (pI > 8.0). Each peptide is described by a series of data points connected by straight lines. Data points corresponded to the scalar descriptor values of the amino acids from the N-terminal (position 1) up to the C-terminal position within the peptide. Two scalar descriptors have been used: the scale developed by Collantes and Dunn (isotropic surface area, ISA and electronic charge index, ECI) and “Volume” and “Hydrophilicity” scale (see Section 4 for details).

As to basic peptides (pI higher than 8.0), four active peptide sequences were available versus seven inactive peptide sequences. The comparison of the line graphs obtained using both scales did not suggest any differential trait related to the hydrophobicity of the amino acid residues between the two groups, in that the scalar descriptor values varied over a wide range from position 1 to position 5 in both active and inactive peptide sequences. On the other hand, the Barley’s volume scalar descriptor varied over a narrow range at positions 2, 3, 4 and 5 of the active peptides, so that the line graphs suggested a peculiar structural feature linked to the molecular size of the amino acid side chains not seen in the inactive peptides: specifically, large amino acid side chains at both positions 2 and 3 followed by small or tiny amino acids at both positions 4 and 5. Finally, as to the Collantes and Dunn scale, the trend of ECI values along the amino acid sequence suggested that a high charge concentration of amino acid side chains at positions 1, 2 and 3 was a preferential character of active peptides, whereas a high charge concentration at positions 4 and 5 was related to a lack of activity. In other words, the presence of L-Arg (not so L-Lys), at or near the N-terminal end may be favorable to CCK-releasing activity, whereas L-Arg near to the C-terminal end makes the peptide basic, but it is unfavorable to CCK-releasing activity (Figure 6).

## 3. Discussion

The knowledge of the structural features underlying the secretagogue effect of protein digestion products on enteroendocrine cells in the gut may help to predict the effects of distinct dietary proteins on this branch of the hormonal control of food intake and energy homeostasis, starting from the current availability of a number of Peptidomics studies with long lists of peptides found in gastrointestinal digestion or among the products of in vitro simulated gastrointestinal digestion of food proteins as well as the availability of bioinformatic tools for the analysis of dietary protein-related full-length amino acid sequences [20,21,22]. This knowledge is still limited, but it would help to design special foods and ingredients producing an adequate metabolic response.

In recent years, articles reporting on the isolation of dietary protein-derived peptide fragments that were able to induce CCK and/or GLP-1 release from enteroendocrine cells in vitro have accumulated. In some studies, the authors also tried to point out some distinctive structural features of these peptides related to their secretagogue activity and to elucidate which receptor might be involved. Briefly, after protein digestion in the gut, both free amino acids and peptides can induce the release of hormones from enteroendocrine cells. Actually, there is evidence supporting the prevailing role of peptidic fragments versus free amino acids, despite it still being under debate [4,5,6,7,8,9,10,11,12,13,14,15,16]. This consideration does not diminish the role played by the free amino acid peak in the plasma after meals in the control of food intake, energy metabolism and anabolic and catabolic functions in tissues, i.e., in skeletal muscle tissue, through multiple direct and indirect actions [3]. Focusing on peptides, the features related to the induction of CCK and/or GLP-1 release may include size, hydrophobicity, net charge, specific amino acid content and the position of the selected amino acid in the chain. It is important to remark that some features may be shared by both CCK- and GLP-1-releasing peptides, but some features may also be distinctive or have been associated with the release of one hormone in experimental studies and cannot be regarded as common to both classes of peptides [1].

A great number of CCK-releasing peptide sequences have been published in the last two decades. If we might want to briefly summarize the conclusions drawn by the authors of related articles, possible distinctive structural features of CCK-releasing peptides include a peptide length in the range 4 to 11 amino acids, an isoelectric point higher than 6.8, high hydrophobicity, and a content of at least one aromatic amino acid. Actually, these characteristics should not be regarded as strictly necessary or, on the other hand, as enough to confer CCK-releasing activity to a peptide. Indeed, a few peptides containing alphatic amino acids in place of aromatic amino acids or peptides endowed with marked hydrophilicity or negative net charge were revealed to be active in vitro. Moreover, despite most of the studies agreeing that a major role is played by peptide size and peptide composition versus specific amino acid sequence motifs in conferring secretagogue activity, in a few studies the removal of one or more selected amino acids from the N-terminal or the C-terminal end of an active peptide was shown to cause a loss of activity without significantly altering the hydrophobicity index or the isoelectric point: specifically, the removal of the N-terminal L-Ser from SRYPS and the removal of YLE from the C-terminus of RYLGYLE [8].

Now, the availability of published CCK-releasing peptide sequences identified and characterized by using quite similar in vitro experimental conditions has suggested the relevance of carrying out a comparative analysis of their primary structure and their physicochemical properties with the aim of finding structural features shared by the secretagogue peptides and, in addition, to highlight possible distinctive features of these peptides compared to peptides that were found to be completely ineffective in the same studies.

The outcome was the fractionation of the available CCK-releasing peptide sequences into distinct subsets according primarily to their size and then to their isoelectric point. Each subset was further analyzed to find the similarities, if any, in the degree of hydrophobicity/hydrophilicity, amino acid composition and amino acid sequence (referring to selected amino acids in selected positions in the chain) between peptides. In this way, for each subset we reported on a combination of structural features which can be regarded as related to CCK-releasing activity in vitro and, where appropriate, we also suggested a reference peptide. Actually, our analysis confirmed the heterogeneity of CCK-releasing peptides from food proteins in their primary structure and their physicochemical characteristics. The relatively lower occurrence of L-Glu and L-Gln in active versus inactive peptides was a striking and unexpected finding.

As mentioned in the Introduction, some factors may account for the heterogeneity of CCK-releasing peptides: i.e., the involvement of multiple distinct nutrient-sensing receptors in mediating the response of enteroendocrine cells to the products of the enzymatic digestion of proteins in both free amino acids and peptides; for each receptor, the possible occurrence of more binding sites for orthosteric and allosteric ligands, with the range of the effective concentrations of peptides as reported in the cited references suggesting low-affinity receptor–ligand interactions.

Before drawing a few conclusions, it is also worth remarking on some limits which are intrinsic to the in vitro research strategy underlying the identification of all the active and inactive peptides mentioned in this study. STC-1 cells are regarded as a valid tool for the investigation of the secretagogue activity of dietary-derived compounds on enteroendocrine cells. Nevertheless, these cells do not express all the receptors and transporters that are believed to play a role in the detection of amino acids and peptides by enteroendocrine cells in the gut wall—so-called nutrient-sensing receptors. Moreover, before being exposed to peptides in buffered balanced salt solution, the cells had long been cultured in growth medium (DMEM). According to the manufacturers, DMEM contains fifteen distinct amino acids, and their concentrations range from 0.1 mM to 0.9 mM except that of glutamine (Q) (over 2 mM). The medium lacks glutamic acid (E), aspartic acid (D), proline (P), alanine (A) and asparagine (N). It would be important to investigate whether the medium composition and the intracellular content of a given amino acid at time 0 may affect the cellular response following exposure to peptides differing in size (di- or tripeptides versus longer peptides) and to peptides endowed with differential amino acid compositions, i.e., high content of aliphatic amino acids or aromatic amino acids or L-glutamine and so on. Furthermore, the fate of a given peptide after being dissolved in aqueous culture medium may affect its biological activities in cells, including secretagogue activity. Peptides may differ in their tendency to form aggregates, from dimers to oligomers, in their low-affinity interactions with the cell surface and adhesion to culture plates and in their passive diffusion, if any, across the lipid bilayer. As to the peptide sequences reviewed in this work, we cannot discuss these issues. We might just add that the predicted hydrophobicity/hydrophilicity and the predicted solubility as defined in PROSO II do not seem to be major determinants in differentiating between CCK-releasing and non-CCK-releasing peptides in vitro.

In vitro analysis is aimed at investigating hormone secretion by enteroendocrine cells in response to direct nutrient sensing, including peptide sensing. In reality, multiple pathways cooperate in the regulation of hormone secretion in vivo. Products derived from food protein digestion can affect the enteroendocrine cell activity acting directly at the apical or basolateral membrane, but they may also play a role after absorption into circulation, acting through multiple indirect pathways as metabolic substrates and precursors of signaling molecules in different tissues. To this end, we have previously mentioned the role played by free amino acid peaks in the plasma after meals. In summary, the ingestion of food proteins is expected to affect the neuroendocrine network underlying energy homeostasis at multiple levels, but direct nutrient sensing may surely have a significant role in the short term.

In conclusion, the currently available set of CCK-releasing peptide sequences and the data on their in vitro activity as reported in the studies reviewed in the present work may not allow a quantitative structure–activity relationship analysis but are fine to use to perform a qualitative analysis of their primary structure and to predict their physicochemical properties using bioinformatics tools. To this end, a method was applied consisting of the fractionation of peptides into subsets and the comparison between paired subsets of active and inactive peptides. For each subset, a few distinctive structural features related to CCK-releasing activity were highlighted. Additionally, the analysis provided evidence that it may be that minor changes in the primary structure make up the difference between active and inactive peptides, as previously suggested by studies addressing the effects of one amino acid change or deletion on CCK- and GLP-1-releasing activity [8]. Hence, the chance to predict the activity of a peptide never tested in vitro using reference structures has to still be considered to be low. Nevertheless, the identification of primary sequence elements related to receptor-mediated biological activity may also be the base for further steps forward addressing the three-dimensional structure of active peptides in molecular docking simulations. This kind of analysis is beyond the aim of the present study, but it could lead to a better understanding of which amino acid changes can be permitted in terms of secretagogue activity. Actually, it is worth remarking that predicting the well-defined spatial structure of short peptides like those included in the third subset of CCK-releasing peptides may be challenging.

Current research on bioactive peptides encrypted in dietary protein sequences aims to exploit the possible functional properties of specific protein sources with different outcomes, including recommendations for healthy diets, development of functional foods and novel dietary supplements with health-related benefits and the development of pharmaceuticals. Success in this applied research field basically depends on technologies enabling large-scale peptide production and delivery systems and increasing peptide bioavailability after oral administration. Current chemical and biotechnological strategies for peptide synthesis and delivery are beyond the aim of this work, and some references may be found elsewhere [4,23]. Briefly, three primary methods are currently used for peptide production: chemical synthesis; enzymatic digestion of proteins using plant, microbial or animal proteases and DNA recombinant technologies. As to improving peptide bioavailability in the gastrointestinal tract, some strategies have been suggested and investigated, including peptide encapsulation and the addition of enhancers for permeation across the intestinal epithelial layer. Focusing on dietary recommendations and claims of health-related benefits of a given food, it is worth remarking that the processing technologies applied to food (i.e., heating techniques in dairy industry), may significantly vary the release of bioactive compounds from a given protein compared to what is expected based on its native structure by altering the digestion rate and the protein susceptibility to proteases or inducing reactions which involve the amino acid side chains. Finally, characterization of the in vitro activity of peptides, peptide isolation and sequencing and bioinformatic analysis of primary peptide structures are necessary steps to conduct applied research in the bioactive peptide field. Then, once peptides of interest have been identified, a more accurate determination of their physicochemical properties by in vitro biochemical analysis is mandatory to optimize large-scale production and delivery. To this end, peptide aggregation and peptide solubility, along with peptide stability, are often a major challenge in peptide application and are difficult to predict. At present, the biochemical characterization of the CCK-releasing peptides discussed in this article was beyond the aim of the study.

## 4. Materials and Methods

### 4.1. Search Strategy and Study Selection

An electronic literature search was performed using the Web of Science, Medline (PubMed) and Scopus online databases. The combination of keywords was as follows: “cholecystokinin (or CCK) secretion” and “inducers” or “CCK-releasing peptides” or “bioactive peptides” and “enteroendocrine cells”. Based on a full text review, we selected original research articles reporting on the characterization of the activity of peptides isolated from peptide mixtures and synthetic peptides on CCK release using STC-1 cell cultures. The peptides sequences extracted from the selected articles were used to prepare two separate sets of peptides: “active peptides”, which induced a significant increase in CCK release versus vehicle-treated cells, and “inactive peptides”, which did not significantly enhance CCK release versus vehicle-treated cells. As additional eligibility criteria, we introduced the following limits: only peptides showing minimal effective concentrations lower than 5 mM were included in the “active peptides” set and only peptides that were ineffective at any concentrations tested were included in the “inactive peptides” set. Finally, this study was not aimed at developing a quantitative structure–activity relationship. Specifically, we considered that it was not possible to compare the quantitative effects of CCK inducers from distinct studies that used different batches of STC-1 cells, due to the possible sizable differences in the basal secretory activity between batches. Lower basal secretory activity may cause an overestimation of the cells’ response to stimuli, even when expressed as relative to vehicle-treated cells. Moreover, STC-1 cells also release GLP-1. To prepare the set of peptides for the analysis, we focused on one enteric hormone, because it has been suggested that the effects of inducers may be hormone-specific. The number of available CCK-releasing peptide sequences was revealed to be higher than that of GLP-1-releasing peptides.

### 4.2. Analysis of Peptide Sequences

In order to search for possible specific features correlating with significant CCK-releasing activity and possible differential features between active and inactive peptides, we basically utilized the frequency of the occurrence of amino acids and peptide sequence alignment strategies. Both sets of peptides were ranked as a function of peptide size and divided into three subsets based on frequency distribution: two to four amino acids, five to eight amino acids and more than eight amino acids. Each subset was analyzed separately.

### 4.3. Description of Physicochemical Properties of Peptides

We used a freely available bioinformatic toolbox (https://www.protpi.ch/calculator/peptidetool) for the calculation of some physicochemical parameters of peptides: monoisotopic mass (m_mono_), isoelectric point (pI) and relative hydrophobicity (H_R_) (access dates from June 2024 to July 2025).

We used https://www.novoprolabs.com/tools/peptide-solubility-pred to predict peptide solubility (access dates in October 2025). This tool is intended for peptides or proteins expressed in *E. coli* that are less than 200 residues long but may also provide more broadly applicable solubility predictions. Solubility was defined in PROSO II as a sequence that was transfectable, expressible, secretable, separable and soluble in the *E. coli* system. The solubility prediction is expressed in terms of probability value (%) as follows: a probability value lower than 40% is unlikely to be soluble, a probability value between 40% and 80% is weakly soluble and a probability value higher than 80% is likely soluble [24].

We used the RasMol Shapely color scheme to color-code the amino acid residues of each peptide according to traditional physicochemical properties related to their side chains based on Robert Fletterick’s “Shapely models”. Finally, we used two distinct sets of scalar descriptors in the attempt to better describe the peptides incorporated in each subset of peptides. The first one was the 3D (conformational) scale developed by Collantes and Dunn, which combines the isotropic surface area (ISA, hydrophobic character of the side chain substituent) and the electronic charge index (ECI, charge concentration of the amino acid) [20]. The second one was the “Volume” and “Hydrophilicity” scale published by Barley et al. [25]. Each peptide was described by a series of data points connected by straight lines. Data points corresponded to the scalar descriptors of the amino acids from the N-terminal (position 1) up to the C-terminal position within the peptide. Each line graph gathered subsets of peptides sharing activity, pI and/or size.

This work was based on a qualitative analysis of peptide sequences which have been previously reported as CCK-releasing peptides in vitro. The analysis included a comparison between these sequences and inactive sequences. Only a few calculations and statistical functions were used. They were mentioned and described in the Section 2, where needed.

## Figures and Tables

**Table 1:** CCK-releasing versus CCK-non releasing peptide sequences: first subset.

**CCK-Rel**							
				**m_mono_**	**pI**	**Sol. (%)**	**Ref.**
**R**	**F**			321	9.1	24	[15]
**P**	**F**	**L**		375	6.0	20	[6]
**R**	**A**	**L**	**G**	415	9.1	53	[8]
**P**	**D**	**L**	**P**	440	3.7	72	[10]
**CCK-NonRel**							
				**m_mono_**	**pI**	**Sol (%)**	**Ref.**
**L**	**L**			244	5.6	40	[9]
**L**	**A**			202	5.6	46	[9]
**L**	**E**	**L**		373	4.1	48	[7]
**L**	**L**	**P**		341	5.4	42	[6]
**I**	**P**	**I**		341	5.6	43	[9]
**I**	**P**	**A**		299	5.6	48	[9]
**V**	**V**	**E**	**P**	442	3.9	56	[7]
**L**	**G**	**M**	**E**	448	4.1	75	[7]
**L**	**K**	**P**	**T**	457	8.6	68	[7]
**E**	**L**	**L**	**K**	501	5.7	52	[7]
**R**	**Y**	**L**	**G**	507	8.3	35	[8]
**L**	**T**	**E**	**Y**	510	3.8	22	[7]

Amino acid sequences of short peptides (first subset: 2 to 4 amino acids long) are shown using one-letter code. Molecular mass (m_mono_), isoelectric point (pI) and solubility prediction (Sol.) are reported. Solubility prediction is expressed in terms of probability value (%). CCK-rel: cholecystokinin-releasing peptides (active peptides); CCK-non-rel: inactive peptides. For each peptide, the reference (Ref.) of the original research article is reported.

**Table 2:** Alignment between a subgroup of CCK-releasing peptide sequences (2 to 4 amino acids long) and the sequence RFYGPV (C-terminal hexapeptide of human osteocalcin, see ref. [19]).

**R**	**F**	**Y**	**G**	**P**	**V**	[19]
**R**	**F**					
**R**	**A**	**L**	**G**			
**P**	**F**	**L**				

**Table 3:** CCK-releasing versus CCK-non releasing peptide sequences: second subset.

**CCK-Rel**																	**m_mono_**	**pI**	**H_R_**	**Sol. (%)**	**Ref.**
**L**	**G**	**A**	**K**	**D**	**S**	**T**	**R**	**T**									947	8.6	5.3	87	[8]
**V**	**L**	**L**	**P**	**D**	**E**	**V**	**S**	**G**	**L**								1040	3.8	31	58	[6]
**V**	**A**	**W**	**R**	**N**	**R**	**C**	**K**	**G**	**T**	**D**							1304	9.1	6.9	92	[8]
**V**	**R**	**I**	**R**	**L**	**L**	**Q**	**R**	**F**	**N**	**K**	**R**	**S**					1685	13	24	53	[14]
**V**	**Y**	**V**	**E**	**E**	**L**	**K**	**P**	**T**	**P**	**E**	**G**	**D**	**L**	**E**	**I**	**L**	1943	3.9	38	68	[5]
**T**	**P**	**E**	**V**	**D**	**D**	**E**	**A**	**L**	**E**	**K**	**F**	**D**	**K**				1635	4	25	66	[5]
**G**	**G**	**V**	**S**	**L**	**P**	**E**	**W**	**V**									942	4.1	31	87	[5]
**F**	**L**	**D**	**D**	**D**	**L**	**T**	**D**	**D**									1067	3.3	25	37	[5]
**K**	**I**	**L**	**D**	**K**	**V**	**G**	**I**	**N**	**Y**	**W**	**L**						1460	8	37	21	[5]
**CCK-NonRel**																					
**G**	**A**	**T**	**G**	**P**	**A**	**G**	**A**	**V**									699	5.6	7.5	74	[7]
**S**	**A**	**G**	**P**	**Q**	**G**	**P**	**I**	**G**	**P**	**R**							1036	9.2	12	89	[7]
**R**	**V**	**A**	**S**	**M**	**A**	**S**	**E**	**K**	**M**								1108	8.3	16	50	[6]
**P**	**Q**	**Q**	**P**	**F**	**F**	**Q**	**P**	**Q**	**Q**	**P**							1340	5.9	25	97	[13]
**Q**	**P**	**Q**	**L**	**P**	**Y**	**Q**	**P**	**Q**	**L**	**P**							1307	5.1	26.2	98	[11]
**R**	**P**	**K**	**H**	**P**	**I**	**K**	**H**	**Q**	**G**	**L**	**P**	**G**					1463	11	7.9	74	[8]
**M**	**E**	**N**	**S**	**A**	**E**	**P**	**E**	**Q**	**S**								1120	3.7	7.3	57	[5]

Amino acid sequences of long peptides (second subset: 9 to 17 amino acids long) are shown using one-letter code. Molecular mass (m_mono_), isoelectric point (pI), relative hydrophobicity (H_R_) and solubility prediction (Sol.) are reported. Solubility prediction is expressed in terms of probability value (%). CCK-rel: cholecystokinin-releasing peptides (active peptides); CCK-non rel: inactive peptides. For each peptide, the reference (Ref.) of the original research article is reported.

**Table 4:** Amino acid composition of CCK-releasing versus CCK-non releasing peptides (second subset).

	CCK-Rel		CCK-NonRel	
		%		%
**R**	7	6.7	3	4.0
**K**	8	7.7	3	4.0
**H**	0	0.0	2	2.7
**F**	3	2.9	2	2.7
**Y**	2	1.9	1	1.3
**W**	3	2.9	0	0.0
**D**	13	12.5	0	0.0
**E**	9	8.7	4	5.3
**N**	3	2.9	1	1.3
**Q**	1	1.0	13	17.3
**S**	4	3.8	5	6.7
**T**	6	5.8	1	1.3
**C**	1	1.0	0	0.0
**M**	0	0.0	3	4.0
**P**	5	4.8	16	21.3
**G**	7	6.7	7	9.3
**A**	3	2.9	7	9.3
**I**	4	3.8	2	2.7
**L**	15	14.4	3	4.0
**V**	10	9.6	2	2.7
**Sum**	104	100	75	100

Moles of each amino acid (first column of CCK-rel and CCK-non rel) and relative amount of each amino acid as a percentage (second column of CCK-rel and CCK-non rel) in the whole second peptide subset (peptide length: 9 to 17 amino acids).

**Table 5:** Alignments of CCK-releasing peptides: second subset, low pI value subgroup.

**V**	**Y**	**V**	**E**	**E**	**L**	**K**	**P**	**T**	**P**	**E**	**G**	**D**	**L**	**E**	**I**	**L**					
								**T**	**P**	**E**	**V**	**D**	**D**	**E**	**A**	**L**	**E**	**K**	**F**	**D**	**K**
								**	**	**		**		**	*	**					
**V**	**Y**	**V**	**E**	**E**	**L**	**K**	**P**	**T**	**P**	**E**	**G**	**D**	**L**	**E**	**I**	**L**					
	**F**	**L**	**D**	**D**	**D**	**L**	**….**	**T**	**D**	**D**											
	*	*	*	*				**		*											
			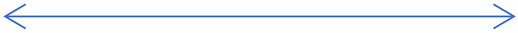																		
**V**	**L**	**L**	**P**	**D**	**E**	**V**	**S**	**G**	**L**												
				**G**	**G**	**V**	**S**	**L**	**P**	**E**	**W**	**V**									
						**	**														
				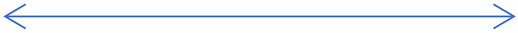																	

Alignments of long CCK-releasing peptides (9 to 17 amino acids long) endowed with low pI values (see Table 2 for details). As to the third alignment, the arrows highlight a suggested scrambled sequence shared by the two peptides. *, amino acids sharing structure similarity.

**Table 6:** Alignment of CCK-releasing peptides: second subset, high pI value subgroup.

**V**	**R**	**I**	**R**	**L**	**L**	**Q**	**R**	**F**	**N**	**K**	**R**	**S**	
**V**	**A**	**W**	**R**	**...**	**...**	**N**	**R**	**C**	**...**	**K**	**G**	**T**	**D**
**			**			*	**			**		*	
**L**	**G**	**A**	**K**	**D**	**S**	**T**	**R**	**...**	**T**				
**V**	**A**	**W**	**R**	**N**	**R**	**C**	**K**	**G**	**T**	**D**			
*	*		*				*		**				
**V**	**R**	**I**	**R**	**L**	**L**	**Q**	**R**	**F**	**N**	**K**	**R**	**S**	
				**L**	**G**	**A**	**K**	**D**	**S**	**T**	**R**	**T**	
				**			*				**	*	
			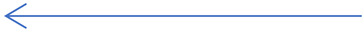										
			**	**	*	**	*						
**K**	**I**	**L**	**D**	**K**	**V**	**G**	**I**	**N**	**Y**	**W**	**L**		
**L**	**G**	**A**	**K**	**D**	**S**	**T**	**R**	**T**					
*	**	*	**	**									
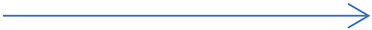													

Alignments of long CCK-releasing peptides (9 to 17 amino acids long) endowed with high pI values (see Table 2 for details). As to the fourth alignment, the arrows highlight a suggested inverted sequence shared by the two peptides. *, amino acids sharing structure similarity.

**Table 7:** CCK-releasing peptide sequences: third subset.

								m_mono_	pI	H_R_	Sol. (%)	Ref.
**I**	**L**	**D**	**K**	**V**				586	5.9	12.07	39	[5]
**V**	**L**	**L**	**P**	**D**				555	4.1	19.86	48	[6]
**P**	**S**	**L**	**V**	**H**				551	7.6	11.12	54	[7]
**D**	**L**	**V**	**D**	**K**				588	4	6.83	49	[7]
**S**	**R**	**Y**	**P**	**S**				608	8.4	5.51	98	[8]
**A**	**L**	**P**	**M**	**H**				567	7.2	12.04	89	[9]
**P**	**H**	**L**	**M**	**A**				567	7.2	12.9	57	[9]
**Y**	**R**	**I**	**V**	**P**	**L**			759	8.1	24.5	43	[10]
**V**	**F**	**L**	**Q**	**P**	**H**			739	7.2	19.69	34	[10]
**R**	**Y**	**I**	**V**	**P**	**L**			759	8.3	26.2	42	[11]
**A**	**A**	**M**	**P**	**L**	**W**			687	5.7	28.15	97	[9]
**Q**	**F**	**D**	**L**	**D**	**D**			751	3.5	18.24	46	[12]
**S**	**F**	**H**	**F**	**P**	**I**			746	6.5	26.92	68	[11]
**I**	**P**	**P**	**L**	**F**	**P**			682	5.4	27.85	43	[11]
**R**	**Y**	**L**	**G**	**Y**	**L**	**E**		912	5.8	27.59	29	[8]
**W**	**I**	**R**	**G**	**C**	**R**	**L**		902	10	22.54	57	[8]
**V**	**P**	**G**	**E**	**I**	**V**	**E**		741	3.8	15.63	57	[5]
**V**	**L**	**D**	**T**	**D**	**Y**	**K**		852	4.2	13.23	38	[5]
**V**	**V**	**T**	**G**	**V**	**G**	**G**	**Q**	715	5.5	8.44	56	[13]
**D**	**R**	**V**	**Y**	**I**	**H**	**P**	**F**	1045	6.9	26.32	25	[9]
**R**	**V**	**T**	**V**	**Q**	**P**	**D**	**S**	900	5.5	9.36	68	[11]
**P**	**A**	**F**	**K**	**E**	**E**	**H**	**L**	969	5.5	19.54	83	[11]

Amino acid sequences of CCK-releasing peptides (third subset: 5 to 8 amino acids long) are shown using one-letter code. Molecular mass (m_mono_), isoelectric point (pI), relative hydrophobicity (H_R_) and solubility prediction (Sol.) are reported. The solubility prediction is expressed in terms of probability value (%). For each peptide, the reference (Ref.) of the original research article is reported.

**Table 8:** CCK-non releasing peptide sequences: third subset.

								m_mono_	pI	H_R_	Sol. (%)	Ref.
**K**	**Y**	**L**	**G**	**L**				592	8.1	19.9	28	[8]
**R**	**G**	**C**	**R**	**L**				603	9.9	6.43	66	[8]
**L**	**L**	**M**	**M**	**K**				634	8.9	21	46	[7]
**L**	**G**	**V**	**D**	**E**				531	3.7	6.84	55	[7]
**E**	**V**	**L**	**S**	**Q**				574	3.9	7.64	60	[7]
**P**	**E**	**E**	**H**	**P**	**T**			708	4.5	3.2	72	[7]
**F**	**F**	**E**	**G**	**P**	**R**			733	4.4	10	49	[12]
**K**	**A**	**H**	**G**	**G**	**V**			567	8.4	3.17	53	[12]
**P**	**F**	**G**	**V**	**G**	**T**			576	6	13.8	56	[10]
**Q**	**I**	**I**	**Q**	**I**	**P**			710	5.1	21.7	47	[10]
**I**	**P**	**A**	**V**	**F**	**K**			673	8.9	19.1	54	[8]
**Y**	**P**	**W**	**Q**	**R**	**F**			895	8.1	27.3	40	[16]
**I**	**F**	**Q**	**P**	**H**	**A**			711	6.8	13.6	25	[11]
**H**	**I**	**H**	**V**	**N**	**G**	**A**		746	6.7	6.87	53	[7]
**E**	**A**	**P**	**L**	**N**	**P**	**K**		767	5.7	9.77	93	[7]
**D**	**S**	**K**	**P**	**G**	**S**	**L**		702	5.8	7.37	82	[7]
**L**	**S**	**Q**	**P**	**F**	**H**	**V**		826	6.8	20.3	44	[11]
**P**	**Q**	**G**	**F**	**A**	**V**	**V**		716	6	20.1	55	[12]
**R**	**A**	**D**	**H**	**P**	**F**	**L**		854	6.4	20.5	38	[6]
**L**	**I**	**V**	**T**	**Q**	**T**	**M**		804	5.6	20.2	37	[8]
**V**	**Q**	**V**	**Q**	**I**	**P**	**F**		829	5.7	24.4	42	[13]
**V**	**A**	**P**	**E**	**E**	**H**	**P**	**T**	878	4.5	6.28	74	[7]
**D**	**V**	**S**	**G**	**G**	**Y**	**D**	**E**	840	3.4	7.07	57	[7]
**F**	**L**	**Q**	**P**	**H**	**Q**	**I**	**A**	952	6.5	22.9	31	[10]
**F**	**R**	**T**	**A**	**T**	**G**	**A**	**V**	821	9.7	13.4	52	[6]

Amino acid sequences of non-CCK-releasing peptides (third subset: 5 to 8 amino acids long) are shown using one-letter code. Molecular mass (m_mono_), isoelectric point (pI), relative hydrophobicity (H_R_) and solubility prediction (Sol.) are reported. Solubility prediction is expressed in terms of probability value (%). For each peptide, the reference (Ref.) of the original research article is reported.

**Table 9:** Amino acid composition of CCK-releasing versus CCK-non releasing peptides (third subset).

	CCK Rel		CCK Non-Rel	
		%		%
**R**	8	5.9	6	3.8
**K**	4	2.9	6	3.8
**H**	7	5.1	9	5.7
**F**	7	5.1	11	6.9
**Y**	7	5.1	3	1.9
**W**	2	1.5	1	0.6
**D**	11	8.1	5	3.1
**E**	5	3.7	9	5.7
**N**	0	0.0	2	1.3
**Q**	4	2.9	12	7.5
**S**	5	3.7	5	3.1
**T**	3	2.2	7	4.4
**C**	1	0.7	1	0.6
**M**	3	2.2	3	1.9
**P**	17	12.5	17	10.7
**G**	6	4.4	14	8.8
**A**	5	3.7	11	6.9
**I**	8	5.9	9	5.7
**L**	17	12.2	13	8.2
**V**	16	11.8	15	9.4
**sum**	136	100	159	100

Moles of each amino acid (first columns of CCK-rel and CCK-non rel) and the relative amount of each amino acid as a percentage (second columns of CCK-rel and CCK-non rel) in the whole peptide subset (peptide length: 5 to 8 amino acids).

**Table 10:** Amino acid motifs (di- and tripeptides) found in the CCK-releasing peptides of the third subset.

**D**	**K**		**Q**	**F**	**D**
**Y**	**K**		**V**	**F**	**L**
**D**	**K**	**V**	**S**	**F**	**H**
**F**	**K**	**E**	**H**	**F**	**P**
			**P**	**F**	
**P**	**H**		**L**	**F**	**P**
**M**	**H**		**A**	**F**	**K**
**V**	**H**				
**P**	**H**	**L**		**A**	**L**
**I**	**H**	**P**	**M**	**A**	
**F**	**H**	**L**	**A**	**A**	**M**
			**P**	**A**	**F**
**P**	**S**		**A**	**A**	
	**S**	**R**			
**P**	**S**	**L**	**F**	**I**	**V**
	**S**	**F**	**W**	**I**	**R**
**D**	**S**		**Y**	**I**	**H**
			**P**	**I**	
**L**	**M**	**A**		**I**	**P**
**P**	**M**	**H**	**R**	**I**	**V**
**A**	**M**	**P**			

The relative content of L-Lys, L-His, L-Ser, L-Met, L-Phe, L-Ala and L-Ile was similar in the active versus the inactive peptides of the third subset. In order to show the flanking amino acids within their active peptide sequences, the C-terminal dipeptides, the N-terminal dipeptides and the tripeptides containing each amino acid are reported. The di- and tripeptide sequences that were also found in the inactive peptide sequences have not been included.

**Table 11:** Alignments of CCK-releasing peptides: third subset, low pI value subgroup.

									m_mono_	pI	H_R_
	**V**	**L**	**L**	**P**	**D**				555	4.1	20
		**D**	**L**	**V**	**D**	**K**			588	4	6.8
**Q**	**F**	**D**	**L**	**D**	**D**				751	3.5	18
			**		**						
			**D**	**L**	**V**	**D**	**K**		588	4	6.8
**V**	**P**	**G**	**E**	**I**	**V**	**E**			741	3.8	16
			*	*	**	*					
**Q**	**F**	**D**	**L**	**D**	…	**D**			751	3.5	18
		**V**	**L**	**D**	**T**	**D**	**Y**	**K**	852	4.2	13
			**	**		**					
			**D**	**L**	........	**V**	**D**	**K**	588	4	6.8
				**L**	**G**	**V**	**D**	**E**	531	3.7	6.8

Alignments of 5- to 8-amino-acid-long CCK-releasing peptides with low pI values (see Table 7 for details). In the fourth alignment, the CCK-releasing peptide DLVDK was compared with the inactive peptide LGVDE (see Table 8). *, amino acids sharing structural similarity.

**Table 12:** Alignments of CCK-releasing peptides: third subset, high pI value subgroup.

**A**											
**S**	**R**	**Y**	**P**	**S**							
	**R**	**Y**	**I**	**V**	**P**	**L**					
	**	**									
**W**	**I**	**R**	**G**	**C**	**R**	**L**					
**Y**	...	**R**	**I**	**V**	**P**	**L**					
*		**				**					
**B**									**m_mono_**	**pI**	**H_R_**
+	**R**	**Y**	**I**	**V**	**P**	**L**			759	8.3	26
-	**K**	**Y**	**L**	........	**G**	**L**			592	8.1	20
	*	**	*			**					
+	**Y**	**R**	**I**	**V**	**P**	**L**			759	8.1	25
-	**Y**	**P**	**W**	**Q**	**R**	**F**			895	8.1	27
	**										
+	**S**	**R**	**Y**	**P**	**S**				608	8.4	5.5
-		**R**	**G**	**C**	**R**	**L**			603	9.9	6.4
		**									
	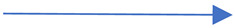										
+	**S**	**R**	**Y**	**P**	**S**				608	8.4	5.5
-	**F**	**R**	**T**	**A**	**T**	**G**	**A**	**V**	821	9.7	13
	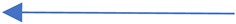				*						
+	**W**	**I**	**R**	**G**	**C**	**R**	**L**		902	10	23
-			**R**	**G**	**C**	**R**	**L**		603	9.9	6.4
			**	**	**	**	**				

Panel (**A**) Alignments of 5- to 8-amino-acid-long active peptides with high pI values (see Table 7 for details). Panel (**B**) Alignments of paired active (+) and inactive (-) peptides. *, amino acids sharing structure similarity.

**Table 13:** Alignments of CCK-releasing peptides: third subset, neutral peptide subgroup.

**CCK-Rel**										
								**m_mono_**	**pI**	**H_R_**
**P**	**H**	**L**	**M**	**A**				567	7.2	13
		**								
**P**	**S**	**L**	**V**	...	**H**			551	7.6	11
		**			**					
**A**	...	**L**	**P**	**M**	**H**			567	7.2	12
		**			**					
**V**	**F**	**L**	**Q**	**P**	**H**			739	7.2	20
		**			**					
**D**	**R**	**V**	**Y**	**I**	**H**	**P**	**F**	746	6.5	27
		*			**					
**S**	**F**	**H**	**F**	**P**	**I**			1045	6.9	26
**CCK-Non-Rel**										
								**m_mono_**	**pI**	**R_H_**
**I**	**F**	**Q**	**P**	**H**	**A**			711	6.8	14
**H**	**I**	**H**	**V**	**N**	**G**	**A**		746	6.7	6.9
**L**	**S**	**Q**	**P**	**F**	**H**	**V**		826	6.8	20
**R**	**A**	**D**	**H**	**P**	**F**	**L**		854	6.4	21
**F**	**L**	**Q**	**P**	**H**	**Q**	**I**	**A**	952	6.5	23

Five- to eight-amino-acid-long peptides endowed with pI values within the range of 6.1 to 7.9. The alignment of the seven active peptide sequences is shown. Molecular mass, isoelectric point and hydrophobicity index are reported. CCK-rel, active peptides; CCK-non-rel, inactive peptides. *, amino acids sharing structure similarity.

**Table 14:** Alignments of CCK-releasing peptides: third subset, mild acidic peptide subgroup.

**CCK-Rel**											
									**m_mono_**	**pI**	**H_R_**
**A**	**A**	**M**	**P**	**L**	**W**				687	5.7	28
	**I**	**P**	**P**	**L**	**F**	**P**			682	5.4	28
	*		**	**	*						
**R**	**Y**	**L**	**G**	**Y**	**L**	**E**			912	5.8	28
**R**	**V**	**T**	**V**	**Q**	**P**	**D**	**S**		900	5.5	9.4
**						*					
**R**	**V**	**T**	…..	**V**	**Q**	**P**	**D**	**S**	900	5.5	9.4
**V**	**V**	**T**	**G**	**V**	**G**	**G**	**Q**		715	5.5	8.4
	**	**		**							
**I**	**L**	**D**	**K**	**V**					586	5.9	12
**V**	**V**	**T**	**G**	**V**	**G**	**G**	**Q**		715	5.5	8.4
*	*			**							
**P**	**A**	**F**	**K**	**E**	**E**	**H**	**L**		969	5.5	20
**CCK-Non-Rel**											
									**m_mono_**	**pI**	**H_R_**
**P**	**F**	**G**	**V**	**G**	**T**				576	6	14
**Q**	**I**	**I**	**Q**	**I**	**P**				710	5.1	22
**E**	**A**	**P**	**L**	**N**	**P**	**K**			767	5.7	9.8
**D**	**S**	**K**	**P**	**G**	**S**	**L**			702	5.8	7.4
**P**	**Q**	**G**	**F**	**A**	**V**	**V**			716	6	20
**L**	**I**	**V**	**T**	**Q**	**T**	**M**			804	5.6	20
**V**	**Q**	**V**	**Q**	**I**	**P**	**F**			829	5.7	24

Five- to eight-amino-acid-long peptides endowed with pI values within the range of 5.1 to 6.0. The alignments of coupled active peptides are shown. Molecular mass, isoelectric point and hydrophobicity index are reported. CCK-rel, active peptides; CCK-non-rel, inactive peptides. *,amino acids sharing structure similarity.

## Data Availability

The original contributions presented in this study are included in the article/Supplementary Materials. Further inquiries can be directed to the corresponding author.

## Supplementary Materials

The supporting information can be downloaded at: https://www.mdpi.com/article/10.3390/ijms262211065/s1.

## Abbreviations

The following abbreviations are used in this manuscript:

CCK	cholecystokinin
m_mono_	monoisotopic mass
pI	isoelectric point
H_R_	relative hydrophobicity
ISA	hydrophobic character of the side chain substituent
ECI	charge concentration of the amino acid
